# PD-L1 expression and the prognostic significance in gastric cancer: a retrospective comparison of three PD-L1 antibody clones (SP142, 28–8 and E1L3N)

**DOI:** 10.1186/s13000-018-0766-0

**Published:** 2018-11-21

**Authors:** Jing Ma, Jianhui Li, Meirui Qian, Weili Han, Miaomiao Tian, Zengshan Li, Zhe Wang, Shuixiang He, Kaichun Wu

**Affiliations:** 1 Department of Gastroenterology, Affiliated Hospital of Xi’an Jiaotong University, Xi’an, 710032 Shaanxi Province China; 2 State Key Laboratory of Cancer Biology and Institute of Digestive Diseases, Xijing Hospital, The Fourth Military Medical University, Xi’an, China; 3 Department of Infectious Diseases, Tangdu Hospital, The Fourth Military Medical University, Xi’an, China; 4 The Pathology Department, The Fourth Military Medical University, Xi’an, China

**Keywords:** Programmed cell death ligand 1, Immunohistochemistry, H-score, Multiplexed immunofluorescence

## Abstract

**Background:**

Immunohistochemistry (IHC) for programmed cell death ligand 1 (PD-L1) displays staining diversity. We compared IHC staining of PD-L1 in gastric cancer (GC) by using three commercially available antibody clones, and analyzed the correlation with the prognosis.

**Methods:**

IHC using PD-L1 antibodies (clones SP142, 28–8 and E1L3N) in 315 formalin-fixed paraffin-embedded samples was qualitatively compared at the 1, 5 and 10% cut-off by two pathologists on total, tumor and immune/stromal cells. We used computer – assisted scoring to quantitatively analyze and compare the “H-score” of PD-L1 expression in 66 samples on total cells. The antibody clone SP142 was selected to investigate the infiltration of PD-L1^+^CD8^+^ T cells using automated quantitative immunofluorescence analyses (*n* = 50) and the prognostic significance. The prognoses were assessed by log-rank test.

**Results:**

PD-L1 clones SP142 and 28–8 displayed great concordance by qualitative (κ = 0.816, 0.810 for total cells and tumor cells at the 5% cut-off) and quantitative analyses (R^2^ = 0.7991, 0.8187 for positive percentage and “H-score”). PD-L1 clone SP142 showed the highest positivity in immune/stromal cells staining (18.41%) compared to 28–8 (7.62%), while clone E1L3N showed poor staining in both tumor and immune/stromal cells. Clone SP142, but not 28–8 and E1L3N, predicted a worse prognosis at the 5% cut-off (*p* = 0.0243). Both the clone SP142 and 28–8 had high inter-pathologist correlation for tumor staining (R^2^ = 0.9805 and R^2^ = 0.9853), but a moderate correlation for stromal/immune cell staining (R^2^ = 0.5653 and R^2^ = 0.5745). Furthermore, a higher density of PD-L1^+^CD8^+^ T cells was correlated with a shorter survival time (R^2^ = 0.0909, *p* = 0.0352).

**Conclusions:**

PD-L1 antibody clone SP142 was superior in cell staining, particularly in immune/stromal cell and prognosis. These findings are important for selection of PD-L1 antibody clones in the future diagnostic test.

**Electronic supplementary material:**

The online version of this article (10.1186/s13000-018-0766-0) contains supplementary material, which is available to authorized users.

## Background

Positive PD-L1 (programmed death-ligand 1) expression by immunohistochemistry (IHC), which has been mainly used to determine PD-L1 status, is a prerequisite for PD-1 blockade therapy. Patients with PD-L1 positive expression show a higher overall response rate than those with PD-L1 negative expression in gastric cancer [1]. Recently, FDA approved the PD-L1 IHC 22C3 PharmDx kit (Dako North America), the PD-L1 28–8 PharmDx kit (Dako North America) and the PD-L1 SP142 Ventana test (Ventana Medical Systems Inc) as a diagnostic test for pembrolizumab, nivolumab and atezolizumab, respectively. Furthermore, PD-L1 antibody clone E1L3N was also used in some anti-PD-1 clinical trials.

There were two problems in the assessment of PD-L1 expression: first, the same antibody clone showed a different staining ability in different tumors and different antibody clones showed the different staining abilities in the same tumor, especially in immune/stromal cells [2–6]. Second, the cut-off value was varied including 1, 5 and 10% cut-offs. PD-L1-positive staining ranges from 17 to 72% in gastric cancer (GC), and this dramatic difference might be due to the use of diverse antibody clones and the lack of a consensus regarding assessment criteria [7–14]. According to the recent clinical trials (NCT01848834 and NCT02335411), 40 to 55% [15] of GCs were PD-L1-positive using the 22C3 monoclonal antibody at a 1% cut-off value (including tumor cells and stromal or immune cells).

Our study aimed to qualitatively and quantitatively compare the expression of PD-L1 on tumor cells and immune/stromal cells in GC using three PD-L1 antibody clones. Five-year overall survival (OS) rates were compared based on the staining patterns of the three antibodies at the 1, 5 and 10% cut-off values. Then, the antibody clone SP142 was selected to characterize PD-L1 expression on CD8^+^ T cells and analyze the correlation with prognosis in formalin-fixed paraffin-embedded (FFPE) tissue samples using quantitatively multiplexed immunofluorescence.

## Methods

### Patients

A consecutive series of 315 surgically resected samples of primary advanced GC was obtained from the Xijing Digestive Hospital from July 1, 2011 to July 1, 2012. All patients were diagnosed with advanced GC (stages I-III) by pathologists based on hematoxylin and eosin (H&E) staining. Patients did not receive any treatment before surgery, while most patients received chemotherapy after surgery. The clinical parameters evaluated for each patient included sex, age, tumor location, depth, differentiation, tumor staging, and vascular and nerve invasion. Tumor staging was based on the 8th American Joint Committee on Cancer (AJCC) criteria. Five-year OS rates were calculated from the date of the first operation to the date of death from any cause or survival 5 years later.

### Immunohistochemistry (IHC)

FFPE tissue specimens were collected from 315 patients, and three 5-μm consecutive sections were cut from each specimen. PD-L1 staining using three primary antibodies was compared: clone SP142 (1:100; Spring Bioscience Corp, rabbit IgG), clone 28–8 (1:300; Abcam, rabbit IgG) and clone E1L3N (1:200; Cell Signaling Technology Inc., rabbit IgG). PD-L1 antibody clone SP142 applied using the Ventana Benchmark platform and clones 28–8 and E1L3N were applied using the Leica Bond platform. Bond™ Epitope Retrieval ER2 Solution and ER1 Solution were used for E1L3N and 28–8 antigen retrieval, respectively. The normal tonsil tissue was used as a positive control and the isotype control was a rabbit IgG monoclonal antibody applied to sections.

### Multiplexed immunofluorescence staining for CD8 and PD-L1

Sections were deparaffinized and rehydrated, antigen retrieval (EDTA pH 9.0) was performed, and endogenous peroxidase activity and nonspecific antigens were blocked. The primary antibody and CD8 cocktail, including PD-L1 (1:100; Spring Bioscience Corp, Clone SP142, rabbit IgG) and anti-human CD8 antibody (1:100; Cell Signaling Technology Inc., Clone C8/144B, mouse IgG1), were co-incubated with the sections overnight at 4 °C. Sections were then simultaneously incubated with two secondary antibodies (1:100; Invitrogen, goat anti-mouse IgG1 cross-adsorbed secondary antibody, Alexa Fluor® Plus 647, and 1:100; Invitrogen, goat anti-rabbit IgG cross-adsorbed secondary antibody, Alexa Fluor® Plus 594). All sections were covered using Fluoroshield containing 4′,6-diamidino-2-phenylindole (DAPI, Abcam) for 10 min at RT to identify nuclei. Normal tonsil tissue was used as a positive control.

### Scoring and evaluation of PD-L1 IHC

Digital images of the PD-L1-stained sections were captured using a digital slide scanner (3DHISTECH, Budapest, Hungary) and analyzed by three independent pathologists and computer-assisted scoring of PD-L1 expression (3DHISTECH, QuantCenter software, Budapest, Hungary). Three pathologists reviewed all slides and indicated the percentage of predominantly membrane PD-L1 staining of any intensity in tumor cells and stromal/immune cells in each high-power field (400×). Tumor cell positivity was scored as the percentage of tumor cells exhibiting membrane staining of any intensity. Immune cell positivity was scored as the proportion of tumor area, including associated intratumor and contiguous peritumor stroma, occupied by PD-L1-stained immune cells at any intensity. Total cell positivity was scored as the percentage of positive cells, including tumor and immune/stromal cells, in all cells. The percentage of tumor cells showing positivity was recorded as less than or equal 1, 5, 10, 25% or greater than 25%, and the percentage of immune/stromal cells showing positive staining was recorded as less than or equal 1% and greater than 1%. All sections were reviewed.

QuantCenter software was then used to quantify the total number, intensity and density of cells expressing PD-L1 in each slide. The software generated an intensity score for each cell, and we defined bins 0–35 as weak positivity, bins 36–53 as median positivity and bins 54–100 as strong positivity. The “H-score” was calculated using “∑pi (i + 1)” for all slides, in which “pi” represented the percentage of positive cells among all cells in the various intensity categories, and “i” represented the staining intensity (*i* = 0, weak positive; *i* = 1, median positive; *i* = 2, strong positive). The “H-score” from the isotype control was subtracted from the PD-L1-stained section to remove the background value.

### Quantitative immunofluorescence staining

A fully integrated imaging system (TissueFAXS, Tissuegnostics) was used to acquire whole-section images. Filters for Texas Red and Cy5 were used for fluorescence excitation with an external light source (X-cite series 120PC Q, Lumen Dynamics). All components were integrated and controlled using the software TissueFAXS (Version 4.1.5140.15 Slides or 4.2 Slides; Tissuegnostics) to function as a fully automated tissue slide scanner. This automated analysis platform was used to quantify the number of single/double positive cells and the positive cell density for the whole tissue sections in an unbiased manner.

### Statistical analysis

The Kappa-Cohen method was used to assess the concordance of each pair of antibody clones. Correlations between the percentage of positive cells and the “H-score” and the “H-score” correlations for each pair of antibody clones were analyzed by calculating Pearson’s correlation coefficients. Five-year OS was estimated using the Kaplan-Meier (K-M) method and compared with the log-rank test. All *p* values were based on two-sided tests, and values less than 0.05 were considered statistically significant. Data were analyzed using SPSS 21.0 for Windows (SPSS Inc., Chicago, IL, USA), and figures were prepared using GraphPad Prism v5.0 for Windows (GraphPad Software, Inc., San Diego, CA, USA).

## Results

### Qualitative and quantitative assessment of PD-L1 antibody clones SP142, 28–8 and E1L3N

**Fig. 1:**
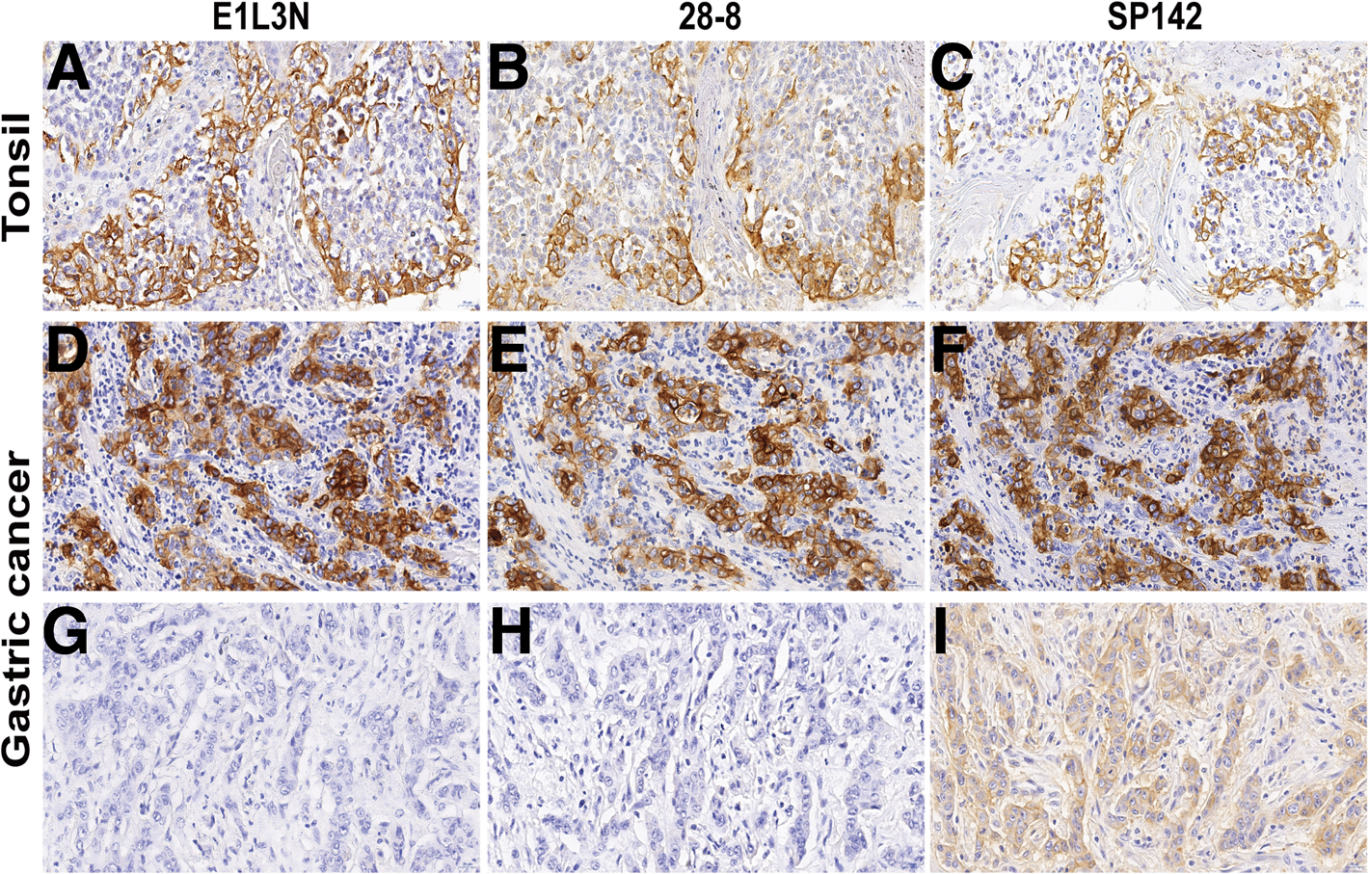
Representative photomicrographs of PD-L1 expression in tonsil tissue and GC. All three antibodies (clone SP142, 28–8 and E1L3N) showed a similar, predominantly membrane staining pattern in tonsil tissues (**a**-**c**) and GC (**d**-**f**). One specimen showed weak positive staining for clone SP142, but negative staining for clones 28–8 and E1L3N (**g**-**i**). The original magnification of all images is 400×. GC, gastric cancer; PD-L1, programmed death ligand 1

**Fig. 2:**
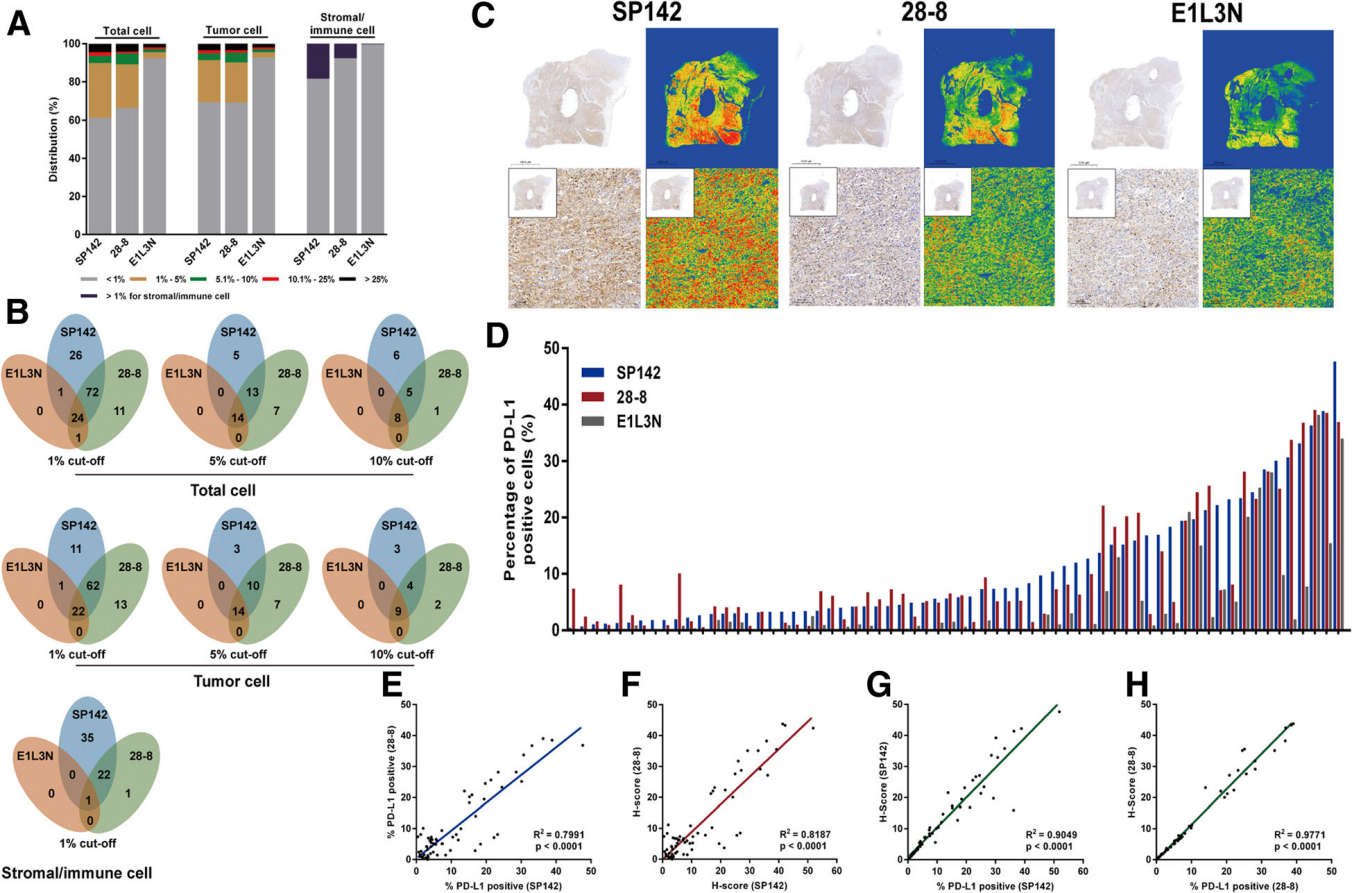
Qualitative and quantitative analysis of PD-L1 staining in total cells, tumor cells and immune/stromal cells. **a** A detailed description of the distribution of PD-L1 expression in total cells, tumor cells and immune/stromal cells stained with clones SP142, 28–8 and E1L3N is shown. **b** A detailed description of the PD-L1 expression at the 1, 5 and 10% cut-off value in total cells and tumor cells, and the 1% cut-off value in immune/stromal cells stained with clones SP142, 28–8 and E1L3N is shown. **c** Images of IHC staining with the three PD-L1 antibody clones and heatmaps analyzing a representative sample showing varying staining intensities and densities for the three clones. **d** A detailed description of the distribution of the PD-L1 expression across three antibody clones for 66 specimens by computer-automated quantitative analysis. Representative plot showing the positive percentage (**e**) and “H-score” (**f**) of total cells positivity for PD-L1 staining using clones SP142 and 28–8 in 66 samples. A strong correlation was observed between clones SP142 and 28–8 (R^2^ = 0.7991 for positive percentage and R^2^ = 0.8187 for “H-score”). **g**, **h** Strong correlations were observed between the percentage of positively stained cells and the “H-score” for clones SP142 (R^2^ = 0.9049) and 28–8 (R^2^ = 0.9771) in 66 samples, particularly clone 28–8. Each dot represents a single specimen. PD-L1, programmed death ligand 1; IHC, immunohistochemistry

Staining of normal human tonsil tissue (Fig. 1a-c) and tumor specimens (Fig. 1d-i) with monoclonal antibody clones SP142, 28–8 and E1L3N revealed specific positive staining for PD-L1 on the cell membrane. The average areas of all reviewed slides were 183.46 mm^2^ for clone SP142 (ranging from 60.80 mm^2^ to 322.20 mm^2^), 184.13 mm^2^ for clone 28–8 (ranging from 62.70 mm^2^ to 350.10 mm^2^), and 183.65 mm^2^ for clone E1L3N (ranging from 65.60 mm^2^ to 335.30 mm^2^). The clinical pathological characters were shown in Additional file 1: Table S1. Two pathologists analyzed PD-L1 expression both on tumor cells and stromal/immune cells (Additional file 2: Figure S1). The distribution of patients in each categorical scoring class for the three assays was described for total cells, tumor cells and stromal/immune cells (Fig. 2a, b). A higher concordance between clones SP142 and 28–8 was observed at the higher cut-off value for total cells (κ = 0.740, 0.816 and 0.823 at 1, 5 and 10% cut-off values, respectively) and tumor cells (κ = 0.813, 0.810 and 0.830 at 1, 5 and 10% cut-off values, respectively). Higher positivity was detected in immune/stromal cells using clone SP142 (58/315, 18.41%) than clone 28–8 (24/315, 7.62%), whereas only one specimen was positive for clone E1L3N (Fig. 2b).

Then, 66 PD-L1-positive specimens were analyzed using a quantitative computer-assisted automated measurement (Fig. 2c, d). We plotted the percentage of PD-L1 expression among total cells and the “H-score” for the antibody clones SP142 and 28–8. The positivity and “H-score” analysis showed a strong correlation between antibody clones SP142 and 28–8 (R^2^ = 0.7991 and R^2^ = 0.8187, Fig. 2E and F, respectively). Furthermore, strong correlations between the “H-score” and the percentage of PD-L1-positive cells were observed for antibody clones SP142 (R^2^ = 0.9049, Fig. 2g) and 28–8 (R^2^ = 0.9771, Fig. 2h).

### Five-year OS analysis at the 1, 5 and 10% cut-off value for PD-L1 antibody clones SP142, 28–8 and E1L3N

**Fig. 3:**
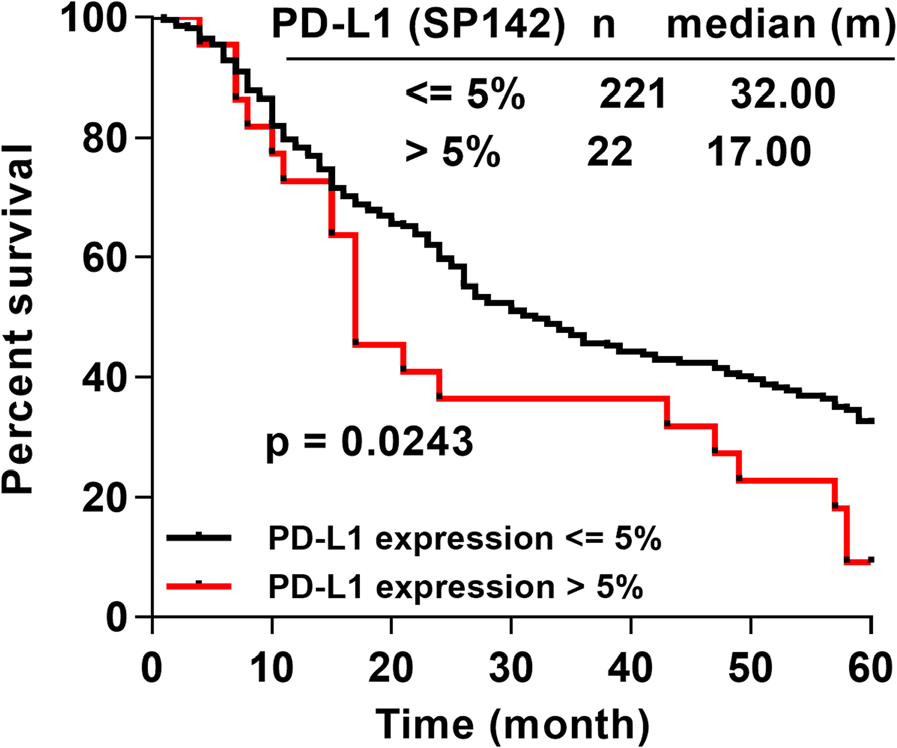
The correlation between five-year OS and PD-L1 expression. The positive expression of the clone SP142 at the 5% cut-off value was correlated with a worse prognosis (log-rank test). “n” represented the numbers of patients displaying staining less than or equal to 5% cut-off value and those displaying staining greater than the 5% cut-off value. OS, overall survival; PD-L1, programmed death ligand 1

The 5-year median survival time (MST) based on PD-L1 expression on total cells, tumor cells and immune/stromal cells was analyzed at the 1, 5 and 10% cut-off values for the three antibody clones. Patients whose total cells displayed SP142 staining at levels greater than the 5% cut-off showed a worse 5-year OS (MST 17.0 m to 32.0 m, HR 1.688, 95% CI of the ratio 1.100 to 3.488, *p* = 0.0243, Fig. 3 and Additional file 3: Figure S2A). Patients whose tumor cells exhibited E1L3N at levels less than the 1% cut-off had a better 5-year OS (MST 32.5 m to 21.0 m, HR 0.5632, 95% CI of the ratio 0.2294 to 0.9349, *p* = 0.0343, Additional file 3: Figure S2B). Meanwhile, immune/stromal cells stained with clones SP142 and 28–8 at levels greater than the 1% cut-off were not significantly different from cells stained at levels less than or equal to the 1% cut-off (Additional file 3: Figure S2C).

### Inter-pathologist correlation for PD-L1 staining for tumor and stromal/immune cells

**Fig. 4:**
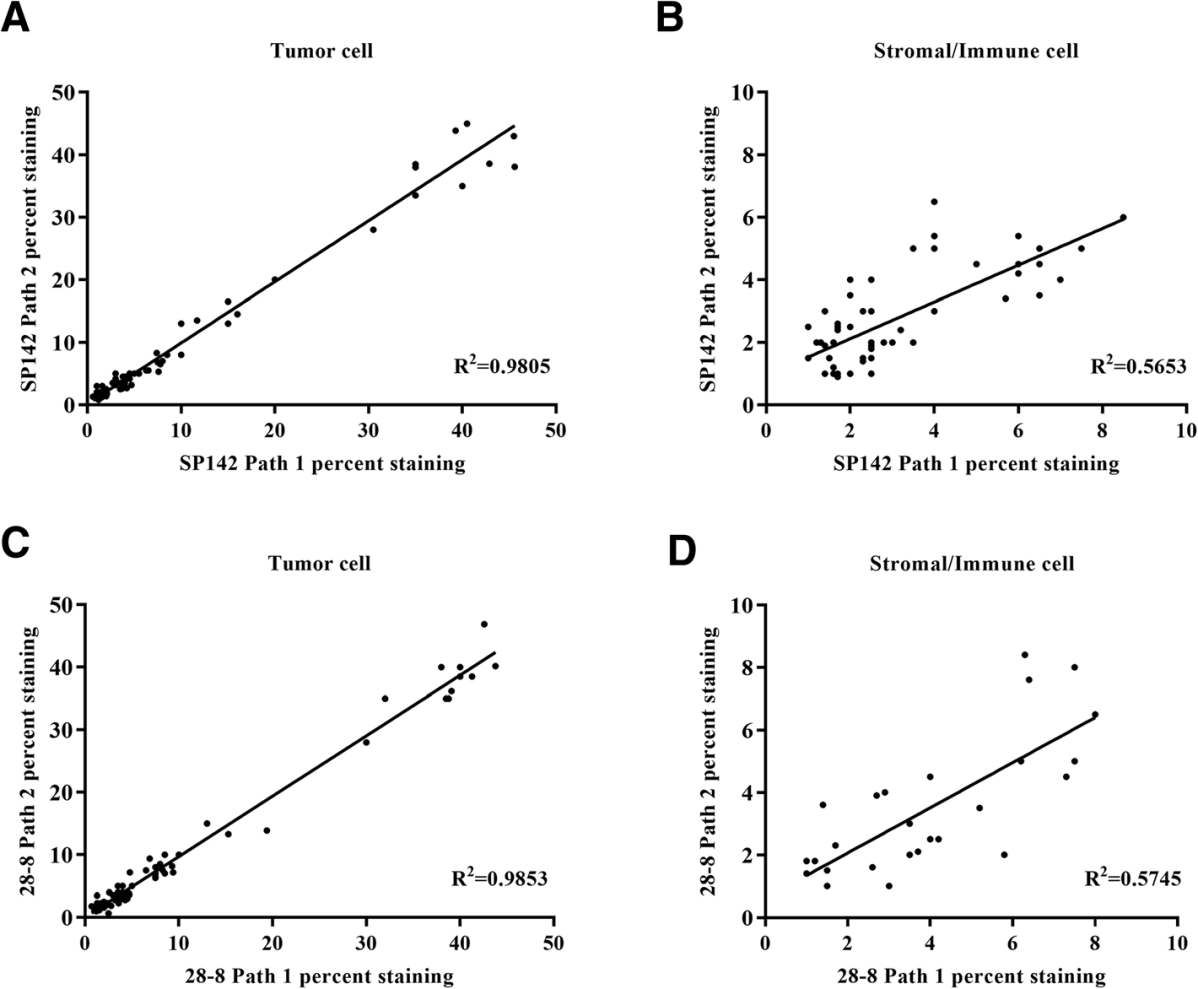
Inter-pathologist correlation of clone SP142 and clone 28–8 PD-L1 expression analysis. Scatter plot comparing the percentage of positive PD-L1 expression in tumor cell (**a**) and stromal/immune cell (**b**) from pathologist 1 and 2 using clone SP142. Scatter plot comparing the percentage of positive PD-L1 expression in tumor cell (**c**) and stromal/immune cell (**d**) from pathologist 1 and 2 using clone 28–8

Both the antibody clone SP142 (R^2^ = 0.9805) and clone 28–8 (R^2^ = 0.9853) had strong inter-pathologist correlation for tumor membrane staining (Fig. 4a, c). However, the correlation was moderate for stromal/immune cell staining using antibody clone SP142 (R^2^ = 0.5653, Fig. 4b) and clone 28–8 (R^2^ = 0.5745, Fig. 4d). These results indicated that the tumor cell staining could be analyzed reproducibly by different pathologists.

### Co-expression analysis of PD-L1 and CD8 in patients with GC using multiplexed immunofluorescence

**Fig. 5:**
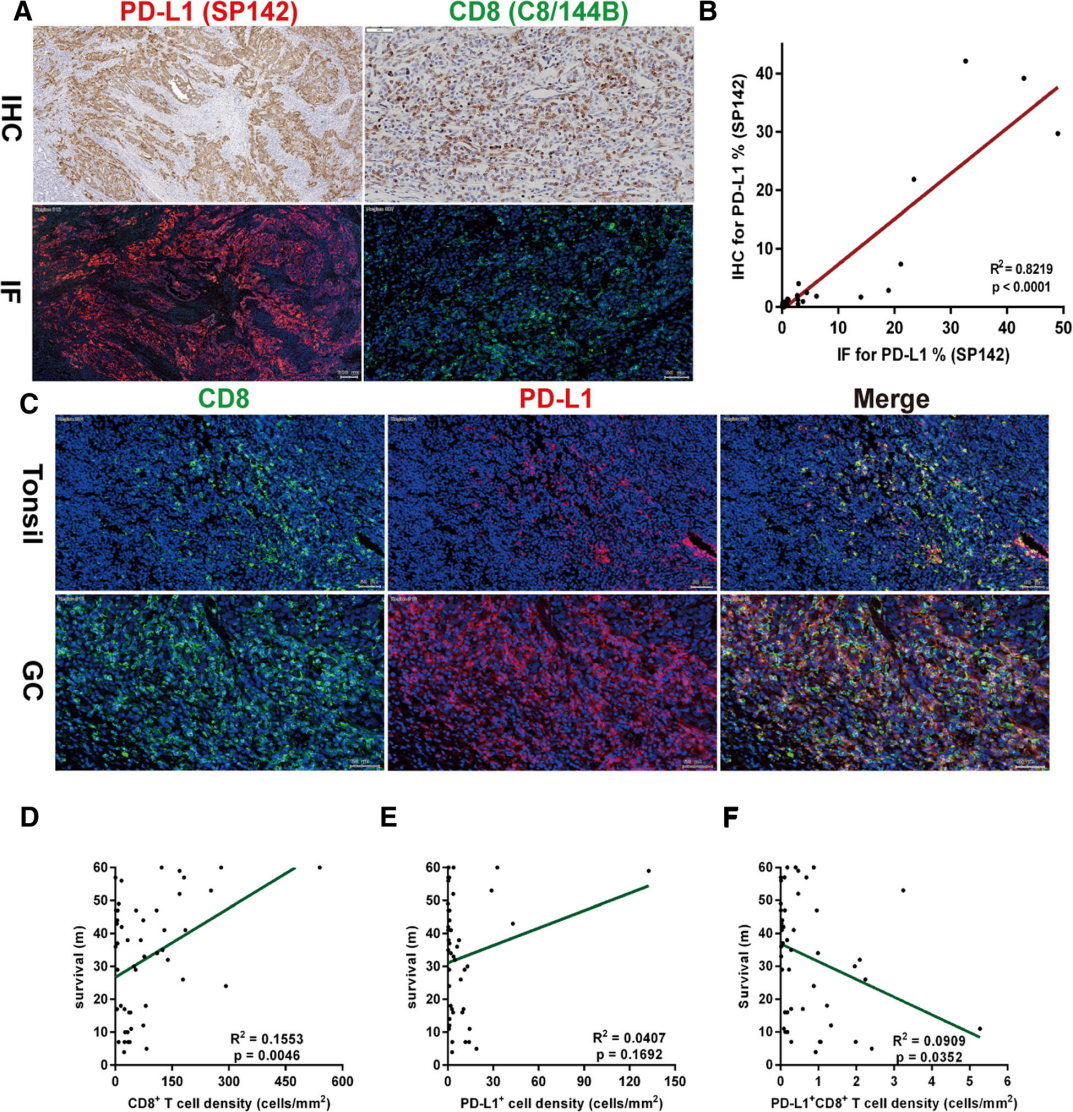
Quantitative analysis of the correlation between density of PD-L1^+^CD8^+^ T cells and survival. **a** Immunofluorescence (IF) for PD-L1 clone SP142 and CD8 clone C8/144B showed an extremely similar staining pattern as IHC, with a strong correlation (R^2^ = 0.8219) for PD-L1 (**b**). Each dot represents a single specimen. Normal tonsil tissue was used as a positive control. **c** Representative images of IF staining in tonsil tissue and GC are shown, in which CD8 was recognized in the Cy5 channel (green) and PD-L1 was recognized in the Texas Red channel (red) and displayed membrane expression. **d** A positive correlation was shown between the density of CD8^+^ T cells and survival time. **e** No relationship was observed between the density of PD-L1^+^ cells and survival. **f** A negative correlation was shown between the density of PD-L1^+^CD8^+^ T cells and survival time. PD-L1, programmed death ligand 1; IF, immunofluorescence; GC, gastric cancer

Different types of immune cells infiltrate into the tumor microenvironment, of which CD8^+^ T cells are essential for killing tumor cells and represent important prognostic indicators. Given the advantage of staining for clone SP142 on immune/stromal cells, we simultaneously characterized the expression of PD-L1 using antibody clone SP142 on CD8^+^ T cells using a multiplexed immunofluorescence analysis of fifty samples. Immunofluorescence staining in the same specimen showed extremely similar tumor cell and the immune/stromal cell staining patterns as IHC for PD-L1 antibody clone SP142 and CD8 antibody clone C8/144B (Fig. 5a). A strong correlation was observed between IHC and immunofluorescence for PD-L1 antibody clone SP142 (R^2^ = 0.8219, *p* < 0.0001, Fig. 5b). Normal tonsil tissue was used as a positive control (Fig. 5c). We analyzed fifty specimens with sufficient fields of view (ranging from 190 to 922) that included tumor cells and immune/stromal cells, and calculated the density of CD8^+^ T cells, PD-L1^+^ cells and CD8^+^PD-L1^+^ T cells. Patients whose tumor showed a higher density of CD8^+^ T cell infiltration exhibited a longer survival time (R^2^ = 0.1553, *p* = 0.0046, Fig. 5d). No correlation was observed between PD-L1 expression and survival (R^2^ = 0.0407, *p* = 0.1692, Fig. 5e). Patients with a higher density of PD-L1^+^CD8^+^ T cell infiltration showed a poorer survival time (R^2^ = 0.0909, *p* = 0.0352, Fig. 5f).

## Discussion

PD-L1 IHC was identified as one approach to select patients who are more likely to respond to anti-PD-1/PD-L1 therapy though “one drug, one assay”, complicating this program (Table 1). Recently, the US Food and Drug Administration (FDA) approved pembrolizumab (Merck) for use in previously treated patients with recurrent locally advanced or metastatic gastric or gastroesophageal junction cancer whose tumors express PD-L1. Thus, the accurate assessment of PD-L1 expression by IHC is essential in clinical practice. This study compared the IHC staining for three commercial PD-L1 antibody clones in 315 FFPE GC specimens. A highly concordant result was observed for clones SP142 and 28–8 in tumor cells, particularly when we used a 5% cut-off value, whereas clone SP142 showed a distinctive advantage in staining immune/stromal cells. Clone E1L3N showed lower levels of positive staining among tumor cells and stromal/immune cells than clones SP142 and 28–8. Pathologists’ evaluation to PD-L1 expression showed a higher concordance in tumor cells than stromal/immune cells. The computer-assisted “H-score” showed the highest concordance with the percentage of positive cells, rather than the staining intensity. Since clone SP142 had the advantage of staining immune/stromal cells, we established a multiplexed immunofluorescence method to quantitatively analyze PD-L1 expression on CD8^+^ T cells using this clone, and found that a higher density of PD-L1^+^CD8^+^ T cells correlated with a shorter survival time.

**Table 1:** The FDA-approved anti-PD1 drug and PD-L1 assessment

mAb	Drug	FDA approval	Scoring assessment	Overall response rate
22C3 pharmDx (Dako North America, Inc.)	Pembrolizumab (KEYTRUDA®)	NSCLC	TPS^a^ < 1%: No PD-L1 expression TPS = 1~ 49%: PD-L1 expression TPS ≥ 50%: High PD-L1 expression	NCT02007070 TPS ≥ 1%:15.4% (95% CI: 4.4–34.9%) TPS ≥ 50%:27.3% (95% CI: 6.0–61.0%)
		Gastric or GEJ adenocarcinoma	CPS^b^ < 1: No PD-L1 expression CPS≥: PD-L1 expression	NCT02335411 CPS ≥ 1: 13.3% (95% CI: 8.2–20.0%)
28–8 pharmDx (Dako North America, Inc.)	Nivolumab (OPDIVO ®)	Melanoma	TC < 1%^c^: No PD-L1 expression TC ≥ 1%^d^: PD-L1 expression	NCT01721746 PD-L1 ≥ 5%: 5.49% (95% CI: 1.92–19.08%) PD-L1 < 5%: 1.13% (95% CI: 0.44–3.16%)
		Non-squamous NSCLC	TC < 1%^e^: No PD-L1 expression TC ≥ 1%^f^: PD-L1 expression	NCT01673867 PD-L1 ≥ 1%: 30.9% (95% CI: 22.9–39.9%) PD-L1 < 1%: 9.3% (95% CI: 4.5–16.4%)
SP142 Assay (VENTANA MEDICAL SYSTEMS, INC)	Atezolizumab (TECENTRIQ)	NSCLC	TC ≥ 50%^g^: PD-L1 expression IC ≥ 10%: PD-L1 expression TC < 50% and IC < 10%^6^: PD-L1 expression	NCT01846416 PD-L1 expression: 16.1% (95% CI:9.32 to 25.2%)
SP263 Assay (VENTANA MEDICAL SYSTEMS, INC)	Durvalumab (IMFINZI™)	Urothelial Carcinoma	TC ≥ 25%: High PD-L1 expression ICP^7^ > 1% and IC + ^7^ ≥ 25%: High PD-L1 expression ICP = 1% and IC+ = 100%: High PD-L1 expression None of the criteria for PD-L1 High Status are met: Low/negative PD-L1 expression	NCT01693562 High PD-L1: 27.6% (95% CI: 19–37.5%) Low/negative PD-L1: 5.1% (1.4–12.5%)

*CI* confidence interval, *CPS* combined positive score, *FDA* Food and Drug Administration, *GEJ* gastroesophageal junction tumor, *ICP* immune cells present, *NSCLC* non-small cell lung cancer, *PD-1*, programmed death receptor 1, *PD-L1* programmed death ligand 1, *TPS* tumor proportion score, *TC* tumor cell

^a^ The percentage of viable tumor cells showing partial or complete membrane staining at any intensity

^b^ The number of PD-L1 staining cells (tumor cells, lymphocytes, macrophages) divided by the total number of viable tumor cells, multiplied by 100

^c^ Specimen is considered PD-L1 negative if < 1% of melanoma cells exhibit circumferential and/or partial linear plasma membrane PD-L1 staining of tumor cells at any intensity. The entire specimen must be evaluated

^d^ Specimen is considered PD-L1 positive if ≥1% of melanoma cells exhibit circumferential and/or partial linear plasma membrane PD-L1 staining of tumor cells at any intensity. The entire specimen must be evaluated

^e^ Non-malignant cells and immune cells (e.g., infiltrating lymphocytes or macrophages) may also stain with PD-L1; however, these should not be included in the scoring for the determination of PD-L1 positivity

^f^ TC are scored as the percentage of tumor cells with the presence of discernible PD-L1 membrane staining of any intensity. IC are scored as the proportion of tumor area, including associated intratumoral and contiguous peritumoral stroma, occupied by PD-L1 staining IC of any intensity

^g^ The percent of tumor area occupied by any tumor-associated immune cells (Immune Cells Present, ICP) is used to determine IC+, which is the percent area of ICP exhibiting PD-L1 positive immune cell staining is also evaluated at any intensity

PD-L1 IHC staining shows staining diversity, namely, the same PD-L1 antibody clone displays different levels of staining in different tumors, and different PD-L1 antibody clones also display different staining patterns in the same tumor. PD-L1 is a transmembrane protein with seven exons. The majority of this protein is located extracellularly, including the binding domain, although it also contains a cytoplasmic domain. PD-L1 staining scores in tumor tissues generally show a lower concordance than in cell lines, and each PD-L1 antibody clone produces different results in different tumor types [16]. Staining for clone SP142 showed lower levels of PD-L1 staining than clones 22C3, 28–8 and E1L3N on immune cells and tumor cells in 90 archival surgically resected NSCLC tumor samples [2], whereas clone SP142 showed a consistent qualitative and quantitative performance for detecting PD-L1 expression in a study comparing staining for five PD-L1 antibody clones (5H1, SP142, 28–8, SP263 and 22C3) in 34 FFPE melanoma specimens [4]. Staining for clone 28–8 showed better consistency in NSCLC, melanoma and triple-negative breast cancer than other antibody clones [4, 5]. Furthermore, staining for clone E1L3N showed the lowest concordance between tumor tissues and cell lines, whereas a high concordance was observed for other clones in nasopharyngeal (NPC), melanoma, NSCLC and breast cancer [4–6, 16]. As shown in the present study, clones SP142 and 28–8 produced more consistent positive staining in tumor cells, suggesting that these two antibodies recognized PD-L1 in a similar manner in tumor cells, although they bind to intracellular and extracellular sites, respectively. In contrast to tumor cells, PD-L1 expression on immune cells showed greater variability, which may have resulted from the distinct staining patterns (both cytoplasmic and membrane), lack of standardized assessment criteria (positive area or positive percentage) and the variable types and levels of stromal cell infiltration [17]. The antibody that binds to the intracellular site (SP142) exhibited better performance than the antibody that binds to the extracellular site (28–8) in staining immune/stromal cells. However, clone E1L3N, which recognizes the intracellular domain, showed weak staining ability in GC. Clone E1L3N may recognize some specific PD-L1 variants, and the status of these variants may impact the staining ability due to secretion, accumulation or degradation [6]. The evaluation of PD-L1 expression in stromal/immune cells showed a moderate consistency by two pathologists. We recommend more pathologists to evaluate the staining effect of stromal/immune cells together. Furthermore, double/triple staining combined PD-L1 with immune cell markers (CD68 for macrophage, CD45 for lymphocytes) may be more precise to evaluate the PD-L1 expression in stromal/immune cells.

The cut-off value, which is used to define patients with positive PD-L1 expression, is controversial in GC. In KEYNOTE-059 cohort 1 and cohort 2, 55% (143/259) and 64% of patients exhibited PD-L1-positive staining using clone 22C3 at the 1% cut-off value, respectively, rates that were higher than those obtained in the current study. Here, 38.73, 33.65 and 7.62% of GC samples were PD-L1-positive at a 1% cut-off value using clones SP142, 28–8 and E1L3N, respectively. These reported differences might be due to the limited sample numbers, the assay characteristics or the binding ability of the antibody itself. A higher proportion of tumor cells were PD-L1-positive than among immune cells using clones SP142 and 28–8 (30.48% vs. 18.41 and 30.79% vs. 7.62%, respectively), which differed from the results reported by Ronan et al., who showed PD-L1 expression in 12% of GC cells and 44% of immune or stromal cells using the clone 5H1 with a 1% cut-off value [18]. Furthermore, we noted a higher concordance at the 5% cut-off value than at the 1% cut-off value in tumor cells stained with antibody clones SP142 and 28–8. Previous PD-L1 expression analyses have not considered the staining intensity. In the present study, we used a computer-automated “H-score” analysis to describe the PD-L1 expression and found that the proportion of positive cells was more closely related to the “H-score” than the intensity.

Elevated levels of PD-L1 expression negatively correlate with OS in several cancers, such as thyroid cancer [19], osteosarcoma [20], triple-negative breast cancer [21] and Epstein-Barr virus-associated GC [22]. In the present study, positive PD-L1 expression (total cells) detected using clone SP142 at the 5% cut-off value was significantly associated with a poor 5-year OS. Low levels of PD-L1 expression (1–5%) might not influence the prognosis. Additionally, PD-L1 expression in stromal/immune cells might influence the function and prognosis. A high level of PD-L1 expression is closely related to the response to PD-1/PD-L1 blockade, particularly when PD-L1 is expressed on tumor-infiltrating lymphocytes [23]. The density and functional status of CD8^+^ T cells are of crucial importance for determining patient survival and the response to immune checkpoint inhibitors. Based on our results, GC tumors with a low level of CD8^+^ T cell infiltration resulted in a poor prognosis, and a greater impairment in CD8^+^ T cell (PD-L1^+^CD8^+^) infiltration indicated worse survival, as determined by multiplexed immunofluorescence. Immunofluorescence-based in situ multiplex measurement of lymphocyte infiltration shows good concordance with flow cytometry in solid tumors [24], consistent with our results obtained using IHC. Clinical trials of drugs blocking PD-1/PD-L1 (NCT02370498, NCT02494583 and NCT03019588) in GC are currently underway, and the relationship between the objective response/clinical benefit and PD-L1 expression by tumor cells and immune cells will be further investigated and discussed.

For the first time, our study reports the qualitative and quantitative comparison of PD-L1 IHC results obtained using three commercial antibody clones (SP142, 28–8 and E1L3N) in GC. Clone 28–8 showed fairly consistent staining on tumor cells, but a lower level of PD-L1 expression on stromal or immune cells than clone SP142. The 5% cut-off value predicted the prognosis better than the 1% cut-off value in specimens stained with clone SP142. Moreover, we observed a correlation between the “exhaustion” status of CD8^+^ T cells and a poor prognosis by analyzing the preliminary outcomes of PD-L1 expression on CD8^+^ T cells using multiplexed immunofluorescence. However, researchers have not determined whether the PD-L1^+^CD8^+^ T cell infiltration influences the response to PD-1/PD-L1 blockade, and our results must be verified in randomized, double-blind and controlled clinical trials in the future.

## Conclusions

PD-L1 antibody clone SP142 was superior in stromal/immune cell staining, and showed a similar staining pattern and positivity with clone 28–8 in tumor cells. The antibody clone E1L3N showed a poor staining in both tumor cells and stromal/immune cells. A higher PD-L1 expression predicted a worse 5-year survival. Furthermore, the higher density of PD-L1^+^CD8^+^ T cells correlated with a shorter survival time. These finding are important for antibody clone selection in the diagnostic test in gastric cancer.

## Additional files

Additional file 1: **Table S1.** Clinical pathological features of patients. (DOCX 19 kb)

Additional file 2: **Figure S1.** A typical micrograph of PD-L1 expression in tumor cell (A) and stromal/immune cells (B). Original magnification × 400. PD-L1, programmed death ligand 1. (TIF 1561 kb)

Additional file 3: **Figure S2.** The correlation between five-year OS and PD-L1 expression in total cells, tumor cells and immune/stromal cells at different cut-off values. The 5-year median overall survival time (MST) was compared among total cells (A) and tumor cells (B) stained with the three antibodies at the 1, 5, and 10% cut-off value, and immune/stromal cells (C) at the 1% cut-off value (log-rank test). “n” represented the numbers of patients displaying staining less than or equal to cut-off value and those displaying staining greater than the cut-off value. OS, overall survival; PD-L1, programmed death ligand 1; MST, median overall survival time. (TIF 2419 kb)

## Abbreviations

AJCC: American Joint Committee on Cancer
CD45: Cluster of differentiation 45
CD8: Cluster of differentiation 8
CPS: Combined Positive Score
FDA: US Food and Drug Administration
FFPE: Formalin-fixed paraffin-embedded
GC: Gastric cancer
H&E: Hematoxylin-eosin staining
HR: Hazard Ratio
IHC: Immunohistochemistry
ORR: Objective response rate
OS: Overall survival
PD-1: Programmed cell death 1
PD-L1: Programmed cell death ligand 1
PFS: Progression-free survival
