# Supplementary material for: PD-L1 expression and the prognostic significance in gastric cancer: a retrospective comparison of three PD-L1 antibody clones (SP142, 28–8 and E1L3N)
Source: Diagn Pathol. 2018 Nov 21;13:91. doi: 10.1186/s13000-018-0766-0 (PMC6249875; doi:10.1186/s13000-018-0766-0)
Supplement: Supplementary file 1 — Table S1. Clinical pathological features of patients. (DOCX 19 kb) [file 13000_2018_766_MOESM1_ESM.docx]

**Table S1. Clinical pathological features of patients**

|  | Total | PD-L1 positive expression | | | |
| --- | --- | --- | --- | --- | --- |
|  |  | SP142 (%)^*^ | 28-8 (%)^*^ | E1L3N (%)^*^ | |
| Age | 315 | 122 | 106 | 24 | |
| Mean (years) | 59.4 | 59.4 | 59.6 | 55.7 | |
| Sex | 315 | 122 | 106 | 24 | |
| Male | 233 (74.0) | 91(74.6) | 81 (76.4) | 16 (66.7) | |
| Female | 82 (26.0) | 31 (25.4) | 25 (23.6) | 8 (33.3) | |
| Tumor location | 312 | 120 | 105 | 24 | |
| Cardia | 79 (25.3) | 33 (27.5) | 30 (28.6) | 6 (25.0) | |
| Body | 60 (19.2) | 31 (25.8) | 27 (25.7) | 7 (29.2) | |
| Antrum | 151 (48.4) | 54 (45.0) | 46 (43.8) | 10 (41.7) | |
| Upper 2/3 | 2 (0.6) | 0 (0.0) | 0 (0.0) | 0 (0.0) | |
| Whole | 20 (6.4) | 2 (1.7) | 2 (1.9) | 1 (4.2) | |
| Differentiation | 285 | 113 | 99 | 23 | |
| High | 19 (6.7) | 8 (7.1) | 7 (7.1) | 1 (4.3) | |
| Moderate | 43 (15.1) | 20 (17.7) | 18 (18.2) | 4 (17.4) | |
| Low | 147 (51.6) | 56 (49.6) | 51 (51.5) | 14 (60.9) | |
| High-Moderate | 16 (5.6) | 0 (0.0) | 1 (1.0) | 0 (0.0) | |
| Low-Moderate | 60 (21.1) | 29 (25.7) | 22 (22.2) | 4 (17.4) | |
| Type | 315 | 122 | 106 | 24 | |
| Adenocarcinoma | 286 (90.8) | 111 (91.0) | 96 (90.6) | 22 (91.7) | |
| Squamous carcinoma | 4 (1.3) | 1 (0.8) | 1 (0.9) | 0 (0.0) | |
| Mucocellulare carcinoma | 8 (2.5) | 2 (1.6) | 2 (1.9) | 0 (0.0) | |
| Others | 17 (5.4) | 8 (6.6) | 7 (6.6) | 2 (8.4) | |
| T | 315 | 122 | 106 | 24 | |
| 1a & 1b | 22 (6.9) | 7 (5.7) | 7 (6.6) | 0 (0.0) | |
| 2 | 39 (12.4) | 14 (11.5) | 13 (12.3) | 3 (12.5) | |
| 3 | 128 (40.6) | 56 (45.9) | 49 (46.2) | 12 (50.0) | |
| 4a & 4b | 126 (40.1) | 45 (36.9) | 37 (34.9) | 9 (37.5) | |
| N | 315 | 122 | 106 | 24 | |
| 0 | 78 (24.8) | 30 (24.6) | 27 (25.5) | 5 (20.8) | |
| 1 | 52 (16.5) | 20 (16.4) | 19 (17.9) | 3 (12.5) | |
| 2 | 50 (15.9) | 17 (13.9) | 14 (13.2) | 4 (16.7) | |
| 3a & 3b | 135 (42.8) | 55 (45.1) | 46 (43.4) | 12 (50.0) | |
| M | 315 | 122 | 106 | 24 | |
| 0 | 315 (100.0) | 122 (100.0) | 106 (100.0) | | 24 (100.0) |
| 1 | 0 (0.0) | 0 (0.0) | 0 (0.0) | 0 (0.0) | |
| AJCC | 315 | 122 | 106 | 24 | |
| ⅠA | 18 (5.7) | 2 (1.6) | 5 (4.7) | 0 (0.0) | |
| ⅠB | 26 (8.3) | 5 (4.1) | 9 (8.5) | 1 (4.2) | |
| ⅡA | 24 (7.6) | 14 (11.5) | 9 (8.5) | 3 (12.5) | |
| ⅡB | 65 (20.6) | 56 (45.9) | 25 (23.6) | 4 (16.7) | |
| ⅢA | 51 (16.2) | 42 (34.4) | 14 (13.2) | 5 (20.8) | |
| ⅢB | 62 (19.7) | 3 (2.5) | 23 (21.7) | 6 (25.0) | |
| ⅢC | 69 (19.7) | 0 (0.0) | 21 (19.8) | 5 (20.8) | |
| Ⅳ | 0 (0.0) | 0 (0.0) | 0 (0.0) | 0 (0.0) | |
| Vascular invasion | 62 (19.7) | 59 (48.8) | 50 (47.6) | 11 (45.8) | |
| Nerve invasion | 69 (19.7) | 47 (39.2) | 39 (37.5) | 9 (37.5) | |
| Survival (m) |  |  |  |  | |
| Median | 30 | 26.5 | 27 | 22.5 | |

^*^: The cut-off value was 1%.
