# Supplementary figures and images for: PD-L1 expression and the prognostic significance in gastric cancer: a retrospective comparison of three PD-L1 antibody clones (SP142, 28–8 and E1L3N)
Source: Diagn Pathol. 2018 Nov 21;13:91. doi: 10.1186/s13000-018-0766-0 (PMC6249875; doi:10.1186/s13000-018-0766-0)

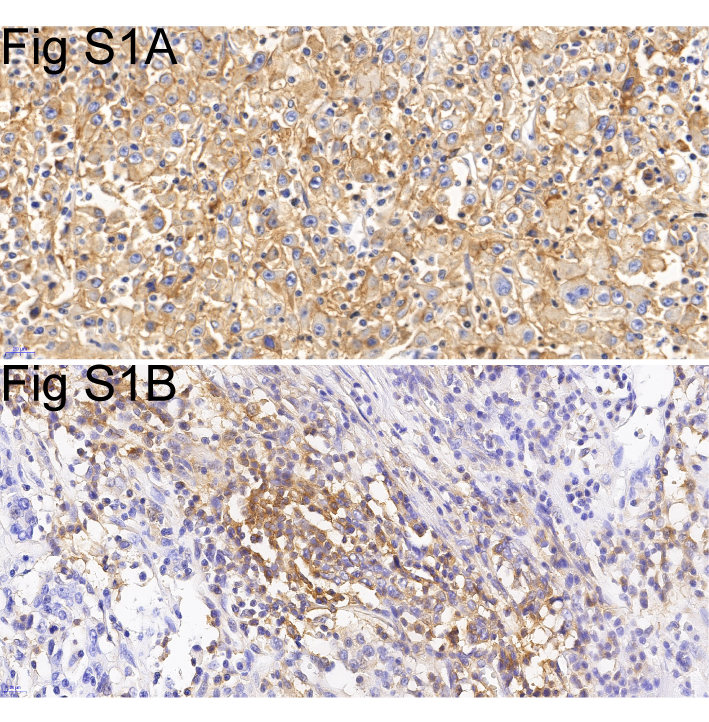

Supplement: Supplementary file 2 — Figure S1. A typical micrograph of PD-L1 expression in tumor cell (A) and stromal/immune cells (B). Original magnification × 400. PD-L1, programmed death ligand 1. (TIF 1561 kb) [file 13000_2018_766_MOESM2_ESM.tif]

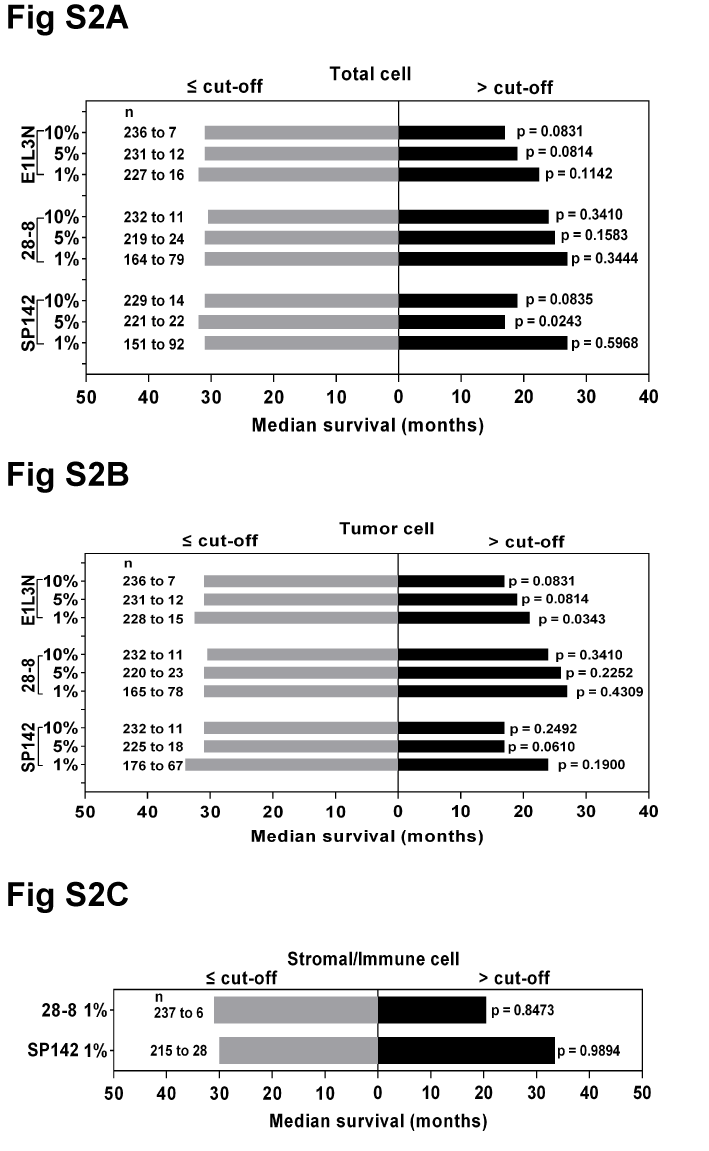

Supplement: Supplementary file 3 — Figure S2. The correlation between five-year OS and PD-L1 expression in total cells, tumor cells and immune/stromal cells at different cut-off values. The 5-year median overall survival time (MST) was compared among total cells (A) and tumor cells (B) stained with the three antibodies at the 1, 5, and 10% cut-off value, and immune/stromal cells (C) at the 1% cut-off value (log-rank test). “n” represented the numbers of patients displaying staining less than or equal to cut-off value and those displaying staining greater than the cut-off value. OS, overall survival; PD-L1, programmed death ligand 1; MST, median overall survival time. (TIF 2419 kb) [file 13000_2018_766_MOESM3_ESM.tif]
